# Quantum molecular resonance electrotherapy (Rexon-Eye) for recalcitrant dry eye in an Asian population

**DOI:** 10.3389/fmed.2023.1209886

**Published:** 2023-09-12

**Authors:** Valencia Hui Xian Foo, Yu-Chi Liu, Bryan Tho, Louis Tong

**Affiliations:** ^1^Ocular Surface Research Group, Singapore Eye Research Institute, Singapore, Singapore; ^2^Corneal and External Eye Disease Service, Singapore National Eye Centre, Singapore, Singapore; ^3^Eye-Academic Clinical Programme, Duke-NUS Medical School, Singapore, Singapore; ^4^Department of Ophthalmology, Yong Loo Lin School of Medicine, National University of Singapore, Singapore, Singapore

**Keywords:** tear disorder, ocular disease, dry eye, therapeutics, clinical trial

## Abstract

**Objectives:**

To assess the safety, efficacy, patients’ satisfaction and acceptability of Rexon-Eye electrotherapy in treating Asian severe dry eye disease (DED) patients.

**Methods:**

Prospective parallel-arm pilot study recruiting 40 DED Chinese patients with >moderate recalcitrant DED (Contact Lens Research Unit [CCLRU] > grade 2). Subjects were randomized into 2 groups, undergoing four weekly treatment sessions each: group 1 received full treatment power; group 2 received control treatment (power 1 treatment). Non-invasive tear break-up time (NIBUT), cornea fluorescein staining graded via CCLRU and Schirmer’s I test were compared pre- and 2 months post-treatment. The SPEED and QUEST questionnaires that evaluated subjective symptoms and treatment satisfaction, respectively, at baseline and 2 weeks post-treatment were carried out. Tear cytokine levels in both groups were examined at 2 weeks post-treatment.

**Results:**

The amount of improvement in post-treatment corneal staining in the inferior corneal zone was significant in Group 1 (*p* = 0.038) but not in Group 2 (*p* = 0.832). Group 1 eyes with worse baseline staining (total score >9.8) had a significantly greater reduction of corneal staining than those with better baseline staining (−11.7 ± 1.98 vs. −4.6 ± 2.89, *p* < 0.001). There were no other significant differences in NIBUT, Schirmer’s 1 and cornea fluorescein staining grading within or between the groups.: Group 1 (*n* = 24) had improved subjective dryness scores compared to Group 2 (*n* = 16) (SPEED score: 6.38 + 4.16 vs. 10.0 + 6.36, *p* = 0.04). No significant differences were seen in 11 tear cytokine levels at 2 weeks post-treatment between the 2 groups.

**Conclusion:**

In Asian DED patients treated with Rexon-Eye, inferior cornea staining showed significant improvement compared to placebo, and eyes with greater cornea staining at baseline achieved a greater improvement in staining. There were no other significant improvements in NIBUT and Schirmer’s 1. Rexon-Eye also improved subjective DED scores in 41.7% of eyes without any adverse effects.

## Highlights

- **What is already known on this topic** - Earlier case studies on Rexon-Eye have shown encouraging improvement in subjective and objective dry eye parameters, but they were mostly conducted small scale in Western populations targeting mild to moderate dry eye disease.
- **What this study adds** - In Asian moderate to severe recalcitrant dry eyes treated with Rexon-Eye, inferior cornea staining showed significant improvement compared to placebo, and eyes with worse corneal staining had the greatest improvement in their ocular surface. Rexon-Eye showed subjective improvement in dry eye symptoms in 40%.
- **How this study might affect research, practice or policy** - Rexon-Eye could be a potential adjunctive treatment option for patients with moderate to severe dry eye disease that is poorly responsive to current treatments.
- **Synopsis/Precis** - In Asian moderate to severe recalcitrant dry eyes treated with Rexon-Eye, inferior cornea staining showed significant improvement compared to placebo, and eyes with worse corneal staining had the greatest improvement in their ocular surface. Rexon-Eye showed subjective improvement in dry eye symptoms in 40%.

## Introduction

Dry eye disease (DED) as defined by the International Dry Eye Workshop (DEWS 2017), is a multifactorial disease of the ocular surface characterized by a loss of homeostasis of the tear film that leads to ocular symptoms due to tear film instability, ocular surface inflammation and neurosensory abnormalities (1). It can be contributed by various factors such as aqueous deficiency or increased tear film evaporation. The available treatments for DED aim to supplement patient’s natural tears or improve the residing time of the tears present (2). Theses include topical artificial tears, ciclosporine, corticosteroids, lifitegrast, punctal occlusion, Microblepharoexfoliation, Thermal Pulsation System and Intense Pulsed Light (IPL) therapy (3). However, these treatments may have various side effects with their use (4); hence low-risk adjunctive DED treatments are often of interest.

Quantum Molecular Resonance (QMR) is a technique in which low-intensity, high-frequency electric currents are administered to a target biological tissue through contact electrodes (5). Earlier studies have proposed several mechanisms of QMR stimulation (6), including mechanical deformation of cellular membrane, transient membrane potential modification and calcium ion release from the sarcoplasmic reticulum. *In vivo* studies showed that a series of cellular contractions and relaxation are then invoked, which triggers cellular metabolism and stimulates tissues downstream. In addition, the expression of matrix metalloproteinase and the infiltration of leukocytes were reduced, supporting an anti-inflammatory effect (5). Electrical stimulation has been hypothesized to affect cell migration and proliferation (7–10), and accelerate nerve regeneration via the promotion of neurotrophic factors such as brain-derived neurotrophic factor and nerve growth factor with the influx of calcium into neurons (11).

Rexon-Eye^®^ (Resono Ophthalmic, Sandrigo, Italy) was developed and patented since 2014 with the basis of stimulating cellular regeneration and reactivating the lacrimal system and tear secretion in DED (6, 12). It uses a broad waveform of 4 to 64 MHz, but with intensities of wavelength within this range optimized for effect on membrane potential and cell regeneration (12). Earlier case studies have been encouraging, reporting improvement in subjective and objective ocular parameters in mixed dry eye patients without adverse effects (6, 13). Other studies also showed its effectiveness in reducing meibomian gland dysfunction (MGD)-induced ocular signs and symptoms (14). Furthermore, previous studies have shown that QMR can accelerate healing in chronic skin wounds (15), reduce pain and muscle injury in inflammatory musculoskeletal diseases (16), and reduce post-operative joint effusion in osteoarthritic knees (17).

However, earlier studies looking at DED-recovery with Rexon-Eye are small scale, targeting mild to moderate DED and conducted mainly in the Western population. Thus, our randomized, masked, comparative interventional study aims to evaluate the safety and efficacy of the Rexon-Eye device in improving recalcitrant DED in an Asian population, while determining patient satisfaction and acceptability of this device.

## Materials and methods

### Design and patient recruitment

This is a randomized comparative, single-center, masked, parallel group, interventional study conducted at the Singapore National Eye Centre from June 2021 to June 2022. Asian patients that attended the Cornea clinic with moderate or worse and recalcitrant DED and met the study inclusion criteria were recruited and randomized into either the full treatment (Group 1) or the control treatment group (Group 2). The study (Clinical trial no: NCT04320563) was approved by the SingHealth Centralized Institutional Review Board (CIRB number: 2019/2446) and complied with the tenets of the Declaration of Helsinki for human research. Informed written consent was obtained from all participants.

### Eligibility

The inclusion criteria were as follows: subjects 21 years of age or older, had at least moderate DED based on grade 2 or worse corneal fluorescein staining graded on the Cornea and Contact Lens Research Unit Grading Scale (CCLRU) grading scale (18) in the central interpalpebral region, had been treated for DED and shown to have no further improvement on maximal therapy (defined as using topical corticosteroids or cyclosporin if medically not contraindicated, and punctal plugs) with no further improvement of symptoms and corneal staining on consecutive visits at least 6 months apart, were already on other topical eye drops (including artificial tears, topical ciclosporin or steroids), lid cleansers or lid warming solely for dry eyes, and with no recent change in topical medications in the last 1 month. Patients were continued on their existing dry-eye medications and were not allowed to change their medication regimen, with the duration of topical medications extending throughout the study period. The exclusion criteria were as follows: contact lens wearers, pregnant women, had active implantable devices (e.g., pacemakers), were oncologic patients undergoing treatment, vegetarian, on topical antibiotic or anti-glaucoma drops, had active ocular infections or eyelid position anomalies.

### Randomization

Consecutive eligible patients were enrolled in this study and were randomly allocated to the study or control group, with a randomization of 3:2, respectively, in order to assess factors of treatment response in the study group. The randomization was conducted by computer-generated random number allocation and applied to sequentially enrolled patients. The randomization schedule was pre-determined, prior to commencing participant recruitment, such that the investigator involved in the baseline participant assessment were not involved in the treatment allocation.

### Study treatment

The treatment is delivered by a device that applies stimulation on the epidermis of closed eyelids up to the lid border via goggles placed directly over the patient’s eyes simultaneously, making the treatment operator-independent. Both groups underwent a session of either the full treatment or comparative treatment per week for 4 weeks, with each session lasting 20 min. Group 1 was assigned to undergo the full treatment power 4, while Group 2 was assigned to undergo the control treatment (low energy, selected as power 1) on the Rexon-Eye device. In order to maximize patient masking, the participants in both groups observed the machine being switched on, but once the mask had been worn, the power setting in the comparative treatment group was reduced to 1.

### Patient evaluation

A questionnaire including demographic and ocular data was completed by all patients at baseline, including age, gender, type and frequency of tear substitutes, additional eye drops or systemic drugs used for DED, any other ocular conditions other than DED or surgeries, and systemic diseases. Dry eye signs and symptoms were evaluated pre- and post-intervention month 2. DED symptoms were assessed using the Standard patient Evaluation for Eye Dryness (SPEED) questionnaire (19).

### Study procedures

Dry eye signs were assessed using Schirmer’s 1 test without anesthesia, non-invasive tear break-up time (NIBUT) (20) and total corneal fluorescein staining via Oculus Keratograph 5 M. The cornea fluorescein staining was scored in 5 corneal zones, central, superior, inferior, nasal and temporal, as in the CCLRU system. In each zone, the grade was assigned 0 to 4, with a greater number indicating a more intense or greater area of staining. The change in corneal staining is calculated as the sum of staining scores in the 5 cornea zones after treatment minus the sum of staining scores in the 5 zones before treatment.

Right at the end of the intervention, the QUEST and Acceptability and Satisfaction Questionnaire (Supplementary Appendix 1) was completed by each subject.

### Tear cytokine elution and analysis

Our tear cytokine procedure has been published and is summarized in Supplementary material (21). Tears were collected from all subjects in the study 2 months after the end of the intervention, in order to assess persistence of any treatment effect 2 months post-treatment.

### Data analysis and sample size calculation

As this therapy has not been evaluated in recalcitrant dry eyes before in a randomized controlled trial, we are unable to base the expected treatment effect on any previous available studies. Based on clincalc.com, assuming the comparative group’s SPEED score to be 10, of the full power treatment group to be 7 or lower, detecting a minimum SPEED difference of 3, with the alpha of 0.05 and power of 80%, we would need 32 subjects overall (or 16 subjects per group). We recruited more (40 subjects) in the event of loss to follow up. Statistical analysis were performed using SPSS version 24. The primary outcomes were the changes in the SPEED questionnaire, and secondary outcomes being Schirmer’s 1 test, NIBUT, corneal fluorescein staining and tear cytokine concentrations pre- and post-intervention. The Wilcoxon signed-rank test was used for intra-group comparisons and the Wilcoxon rank-sum test was used for inter-group comparisons. Data are presented as the mean + standard deviation (SD) unless otherwise stated. All tests were two-tailed, and *p* < 0.05 was considered significant.

## Results

Forty Chinese patients who met the inclusion criteria were included in the study, with 24 participants in the study group and 16 participants in the control group. Overall mean age was 66.55 + 10.45 years, There were no significant differences in age or gender between the two groups. Before treatment, 40% had undergone previous refractive or cataract surgery. Baseline SPEED questionnaire scores were not significantly different between both groups. There were also no significant differences in the baseline Schirmer’s 1 test, NIBUT and corneal fluorescein staining in all 5 zones. Schirmer’s 1 test results were slightly worse in the left than right eye of the subjects, (2.64 + 4.71 vs. 3.33 + 5.51), while NIBUT was slightly worse in the right than the left eye of the subjects (5.05 + 3.04 vs. 5.63 + 4.24). The inferior corneal zone had the highest grade of corneal staining amongst all 5 quadrants in both groups (Table 1).

**TABLE 1:** Clinical and demographic characteristics and baseline DED parameters of participants.

	Overall Mean + SD/*n* (%)	Group 1 full power Mean + SD/*n* (%)	Group 2 comparative^†^ Mean + SD/*n* (%)	*p*-value*
Total number	40	24	16	
Female	36 (90.0)	21 (87.5)	15 (93.8)	0.67
Age (years)	66.55 + 10.45	64.08 + 11.0	70.25 + 8.49	0.07
**Current medications**				
Topical Ciclosporin	16 (40.0)	8 (33.3)	8 (50.0)	0.87
Topical steroids	13 (32.5)	6 (25.0)	7 (43.8)	0.79
Topical Diquafosol	11 (27.5)	6 (25.0)	5 (31.3)	0.65
Schirmer (mm), right eye	3.33 + 5.51	3.41U5.8	3.21 + 5.25	0.70
Schirmer (mm), left eye	2.64 + 4.71	2.45 + 4.62	2.93 + 5.0	0.57
NIBUT^††^ (s), right eye	5.05 + 3.04	5.43 + 3.49	4.46 + 2.18	0.12
NIBUT^††^ (s), left eye	5.63 + 4.24	5.98 + 4.92	5.09 + 2.99	0.29
**Fluorescein staining^†††^, right eye**				
Superior	0.86 + 1.27	0.83 + 1.21	0.91 + 1.39	0.67
Inferior	3.06 + 1.08	3.06 + 1.14	3.06 + 1.03	0.67
Nasal	2.50 + 1.34	2.58 + 1.25	2.38 + 1.50	0.26
Temporal	1.94 + 1.26	1.83 + 1.38	2.09 + 1.20	0.64
Central	1.44 + 1.29	1.50 + 1.23	1.34 + 1.40	0.43
**Fluorescein staining^†††^, left eye**				
Superior	0.91 + 1.36	0.67 + 1.20	1.28 + 1.53	0.09
Inferior	3.14 + 1.23	3.13 + 1.36	3.16 + 1.04	0.25
Nasal	2.66 + 1.38	2.62 + 1.42	2.72 + 1.35	0.78
Temporal	1.66 + 1.55	1.58 + 1.49	1.78 + 1.68	0.31
Central	1.45 + 1.51	1.42 + 1.51	1.50 + 1.56	0.80
SPEED questionnaire	9.03 + 5.19	8.13 + 4.12	10.31 + 8.30	0.24

^†^Participants are classified under dry eye if they demonstrated dry eye symptoms and one of the clinical signs (Staining, Schirmer’s test, or NIBUT). Since participants in the dry eye group tend not to have abnormal results in all the tests: staining, Schirmer’s test and NIBUT, these parameters are individually not lower than the control group, and the values had a large SD. ^††^Non-invasive tear break up times. ^†††^The staining were mild in the zones when present, with no cases of above 10 fluorescein spots in any single corneal zone. Control and dry eye groups were not significantly different in any of the parameters above (*p* > 0.05). *Based on independent sample *t*-tests or Wilcoxon rank-sum test, comparing characteristics between the two groups.

Because this study was conducted on participants on maximal DED standard therapy, and participants continued their use of topical therapy during the study period, we report the use of concurrent medications in each of the two study groups (Table 1). There were no significant differences in the number of subjects on topical ciclosporin, Diquafosol or steroids between the 2 groups at baseline (*p* > 0.05).

On the study visit 2 weeks after the last treatment, the SPEED questionnaire scores significantly improved from baseline in both groups 1 and 2 (Table 3), with group 1 having significantly lower scores than the group 2. (6.38 + 4.16 vs. 10.0 + 6.36, *p* = 0.04) (Table 2). Within group 1, majority of the subjects (58.3%) had maintained scores, while 41.7% had improved scores. Within group 2, interestingly, 64.3% had improved scores, while 35.7% had maintained scores. None had worsened scores in either groups (Table 4).

**TABLE 2:** Main outcomes post-treatment.

	Overall Mean + SD/*n* (%)	Group 1 full power Mean + SD/*n* (%)	Group 2 comparative^†^ Mean + SD/*n* (%)	*p*-value*
Total number	40	24	16	
Schirmer (mm), right eye	3.49 + 4.26	3.22 + 5.21	4.0 + 5.56	0.68
Schirmer (mm), left eye	2.77 + 4.15	3.13 + 4.62	2.08 + 3.12	0.49
NIBUT^††^ (s), right eye	6.01 + 4.23	6.54 + 5.01	5.15 + 2.39	0.26
NIBUT^††^ (s), LE	5.72 + 4.38	5.96 + 3.73	5.33 + 5.41	0.67
**Fluorescein staining^†††^, right eye**				
Superior	0.75 + 1.21	0.75 + 1.18	0.71 + 1.28	0.93
Inferior	2.57 + 1.43	2.42 + 1.54	2.82 + 1.23	0.41
Nasal	2.14 + 1.55	2.29 + 1.54	1.89 + 1.58	0.45
Temporal	1.33 + 1.37	1.46 + 1.41	1.11 + 1.32	0.45
Central	1.26 + 1.41	1.38 + 1.48	1.07 + 1.31	0.53
**Fluorescein staining, ^†††^, left eye**				
Superior	0.81 + 1.56	0.69 + 0.98	1.18 + 1.51	0.23
Inferior	2.87 + 1.23	2.75 + 1.35	3.07 + 1.22	0.47
Nasal	2.61 + 1.65	2.69 + 1.41	2.57 + 1.40	0.81
Temporal	1.71 + 1.31	1.54 + 1.46	1.86 + 1.40	0.52
Central	1.56 + 1.31	1.48 + 1.31	1.68 + 1.50	0.67
SPEED questionnaire	**7.65 + 5.21**	**6.38 + 4.16**	**10.0 + 6.36**	**0.04**

^†^Participants are classified under dry eye if they demonstrated dry eye symptoms and one of the clinical signs (Staining, Schirmer’s test, or NIBUT). Since participants in the dry eye group tend not to have abnormal results in all the tests: staining, Schirmer’s test and NIBUT, these parameters are individually not lower than the control group, and the values had a large SD. ^††^Non-invasive tear break up times. ^†††^The staining were mild in the zones when present, with no cases of above 10 fluorescein spots in any single corneal zone. *Based on the independent sample *t*-test, comparing characteristics between the two groups. Bolded values are those with significant *p* values.

**TABLE 3:** Pre- vs. post-treatment paired *T*-test *P*-values within each group.

		Pre vs. post paired *T*-test *p*-values	
		**Group 1 full** **power**	**Group 2** **comparative**
**Speed**		0.031*	0.008*
NIBUT	Right eye	0.259	0.353
	Left eye	0.905	0.978
Schirmers	Right eye	0.789	0.407
	Left eye	0.497	0.638
Staining right	Superior	0.505	0.067
	**Inferior**	0.038*	0.832
	Nasal	0.252	0.927
	**Temporal**	0.310	0.005*
	Central	0.703	0.551
Left	Superior	0.448	0.823
	Inferior	0.333	0.713
	Nasal	0.414	0.608
	Temporal	0.918	0.820
	Central	0.419	0.111

Bolded values are those with significant *p* values.

**TABLE 4:** Change in outcomes post-treatment.

	Group 1 full power (*n* = 24) *n* (%)	Group 2 comparative (*n* = 16) *n* (%)	*P*-value*
Schirmer (mm), right eye			0.84
Improved	7 (29.2)	7 (43.8)	
Same	13 (54.2)	7 (43.8)	
Worse	4 (16.6)	2 (12.4)	
Schirmer (mm), left eye			0.43
Improved	8 (33.3)	4 (25.0)	
Same	13 (54.2)	8 (50.0)	
Worse	3 (12.5)	4 (25.0)	
NIBUT^††^ (s), right eye			0.75
Improved	10 (41.8)	8 (50.0)	
Same	4 (16.4)	3 (18.8)	
Worse	10 (41.8)	5 (31.3)	
NIBUT^††^ (s), left eye			0.83
Improved	11 (45.8)	6 (37.5)	
Same	3 (12.5)	2 (12.5)	
Worse	10 (41.7)	8 (50.0)	
Fluorescein staining in inferior corneal zone^†††^, right eye			0.82
Improved	9 (37.5)	7 (43.8)	
Same	11 (45.8)	6 (37.5)	
Worse	4 (16.7)	3 (18.8)	
Fluorescein staining in inferior corneal zone^†††^, left eye			0.78
Improved	8 (33.3)	5 (31.3)	
Same	11 (45.8)	9 (56.3)	
Worse	5 (20.8)	2 (12.5)	
SPEED questionnaire			0.12
Improved	10 (41.7)	10 (62.5)	
Same	14 (58.3)	6 (37.5)	
Worse	0	0	

^††^Non-invasive tear break up times. ^†††^The staining were mild in the zones when present, with no cases of above 10 fluorescein spots in any single corneal zone. *Based on independent sample *t*-test, comparing characteristics between the two groups.

The overall post-treatment mean Schirmer’s 1 test improved compared to pre-treatment, however, they were not significantly different between or within each group pre- and post-treatment in either eye (Table 3). We also observed that a greater proportion of left eyes in group 1 had stable or improved Schirmer’s 1 compared to the left eyes in group 2 post-treatment (87.5 vs. 75.0%, *p* = 0.43), although this difference was not statistically significant. In the right eyes, there were similar proportions of stable or improved Schirmer’s 1 between both groups (81.8 vs. 83.4%, *p* = 0.84) (Table 4).

Overall NIBUT also improved in both groups post- compared to pre-treatment, and group 1 had greater NIBUT than the group 2, although this differences between and within each group pre- and post-intervention were not statistically significant (Table 3). However, we noted the right eyes had a lower proportion of stable or improved NIBUT in group 1 compared to group 2 (right eye: 58.2 vs. 68.8%, *p* = 0.75), and the left eyes had a greater proportion of stable or improved NIBUT in group 1 compared to group 2 (left eye: 58.3 vs. 50%, *p* = 0.83) (Table 4).

In terms of corneal staining, the overall mean grades improved post- compared to pre-treatment. The inferior zone of group 1 (right eye) had a significant reduction in staining compared to baseline (*p* = 0.038) whereas the temporal zone of group 2 had a significant reduction in staining compared to baseline (Table 3). Group 1 also had lower staining grading than the Group 2 in the inferior corneal zones for both right and left eyes (right eye: 2.42 + 1.54 vs. 2.82 + 1.23, *p* = 0.41, left eye: 2.75 + 1.35 vs. 3.07 + 1.22, *p* = 0.47) (Table 4), although this difference was not statistically significant. Majority of both right and left eyes had improved or maintained staining in their inferior zones in both groups 1 and 2 (right eye: 83.3 vs. 81.3%, *p* = 0.82, left eye: 79.1 vs. 87.6%, *p* = 0.78) (Table 4).

When we divided group 1 according to their pre-treatment severity of corneal staining, the eyes with more severe staining (total score >9.8 or mean) resulted in significantly greater reduction of corneal staining than the eyes starting with less severe staining (total score <9.8 or mean) (−11.7 ± 1.98 and −4.6 ± 2.89, *p* < 0.001) (Results not shown in Tables).

Analysis of 11 different cytokine tear levels showed no significant differences between groups 1 and 2 at baseline (all *p* > 0.05, Table 5). Analysis of the cytokine tear levels post-treatment also showed no statistically significant differences in all tear cytokine levels between groups 1 and 2, and in all tear cytokine levels pre- and post- treatment (all *p* > 0.05, Table 5). Pre- and post-treatment cytokine tear levels within either groups were also not significantly different (all *p* > 0.05, not shown).

**TABLE 5:** Tear cytokines concentration pre- and post- therapy between groups (pg/ml).

		Group 1 full power		Group 2 comparison		
		**Mean**	**SD**	**Mean**	**SD**	*p*-value*
GM-CSF	Pre	4.85	5.34	5.05	4.42	0.91
	Post	4.36	4.96	4.00	3.27	0.80
IFNg	Pre	60.36	78.53	66.94	81.29	0.83
	Post	58.43	57.55	50.16	64.45	0.71
IL-1B	Pre	47.31	99.59	69.17	111.45	0.58
	Post	67.40	125.77	61.68	146.16	0.91
IL-2	Pre	6.00	7.01	4.69	4.21	0.51
	Post	7.50	12.96	5.39	4.97	0.50
IL-4	Pre	5.57	5.34	5.20	3.55	0.82
	Post	5.20	3.22	4.96	3.05	0.83
IL-5	Pre	27.49	25.25	24.30	17.87	0.68
	Post	19.28	16.28	19.06	16.48	0.97
IL-6	Pre	132.11	148.87	263.61	278.22	0.19
	Post	418.66	595.26	213.90	277.36	0.19
IL-8	Pre	1667.97	2877.72	2160.64	1842.07	0.55
	Post	6593.51	11688.54	4291.61	9006.89	0.53
IL-10	Pre	6.57	6.69	7.02	6.72	0.86
	Post	5.90	6.20	5.38	4.63	0.78
IL-12p70	Pre	13.25	17.39	10.29	8.58	0.52
	Post	15.96	30.08	9.17	7.35	0.32
TNF-a	Pre	14.97	18.99	26.98	31.81	0.25
	Post	16.10	17.38	21.73	32.70	0.59

*Based on independent sample *t*-test, comparing characteristics between the two groups.

Majority found the treatment pleasant compared to the comparative treatment (83.3 vs. 57.1%, *p* = 0.045). The rest of the results were not statistically significant between both groups (Table 6). A total of 37.5% of subjects felt the treatment was effective in group 1 compared to 21.4% in group 2, and 29.2% in group 1 felt the treatment was durable compared to 14.3% in group 2. A total of 41.7% also felt the treatment was better than other DED treatments in group 1 compared to 21.4% in group 2. Overall, 41.2% had felt that their DED situation was improved in group 1 compared to 35.7% in group 2. However no significant difference in the frequency of tear substitutes usage were observed between the groups, and close to 100% still had dry eye symptoms and maintained the same number of daily tear substitutes eye drops at the end of treatment in both groups (Table 6). No adverse effects related to the use of Rexon-eye occurred, such as increased intraocular pressure, worsened visual acuity, or increased discomfort after the intervention.

**TABLE 6:** Change in satisfaction and acceptability post-treatment at 2 months.

*n* (%)	Group 1 full power	Group 2 comparative	*P*-value*
Situation compared to before treatment			0.46
Better	12 (50)	9 (64.0)	
Same	10 (41.7)	5 (35.7)	
Worse	2 (8.3)	2 (14.3)	
Situation compared to end of treatment			0.38
Better	11 (45.8)	9 (64.0)	
Same	11 (45.8)	5 (35.7)	
Worse	2 (8.3)	2 (14.3)	
Situation compared to 1 month after treatment			0.46
Better	10 (41.7)	5 (35.7)	
Same	12 (50.0)	9 (64.0)	
Worse	2 (8.3)	2 (14.3)	
Dry eye symptoms during last week			0.90
Yes	22 (91.7)	13 (92.9)	
No	2 (8.3)	1 (7.1)	
Used lubricants in last 1 week during day			0.60
Yes	24 (100.0)	14 (100.0)	
No	0	0	
Used lubricants in last 1 week during night			0.58
Yes	21 (87.5)	13 (92.9)	
No	3 (12.5)	1 (7.1)	
In last 2 months had other eye problems			0.19
Yes	0	1 (7.1)	
No	24 (100.0)	13 (92.9)	
Last 2 months used lubricants			–
Yes	24 (100.0)	14 (100.0)	
No	0	0	
Last 2 months tried to suspend lubricants			0.44
Yes	1 (4.2)	0	
No	23 (95.8)	14 (100.0)	
Eyes now have more tears			0.97
Yes	7 (29.2)	4 (28.6)	
No	17 (70.8)	10 (71.4)	
Eyes now more wet			
Yes	7 (29.2)	6 (42.9)	0.74
No	17 (70.8)	9 (64.3)	
Eyes feel better than before treatment			
Yes	14 (58.3)	8 (57.1)	0.94
No	10 (41.7)	6 (42.9)	
Use less lubricants now than before Treatment			0.15
Yes	4 (16.7)	1 (7.1)	
No	20 (83.3)	13 (92.9)	
Pleasant			**0.045**
Yes	**20 (83.3)**	**8 (57.1)**	
No	0	0	
Neutral	4 (16.7)	6 (42.9)	
Effective			0.09
Yes	9 (37.5)	3 (21.4)	
No	7 (29.2)	2 (8.3)	
Neutral	8 (33.3)	9 (64.3)	
Durable			0.22
Yes	7 (29.7)	2 (14.3)	
No	7 (29.7)	4 (28.6)	
Neutral	10 (41.7)	8 (58.7)	
With respect to other treatments			0.29
Better	10 (41.7)	3 (21.4)	
Same	13 (54.2)	11 (78.6)	
Worse	1 (4.1)	0	

*Based on the independent sample *t*-tests, comparing characteristics between the 2 groups. Bolded values are those with significant *p* values.

## Discussion

In our prospective study of Asian recalcitrant dry eye patients treated with Rexon-Eye, inferior cornea staining showed significant improvement compared to placebo, and eyes with greater cornea staining at baseline achieved a greater improvement in staining. There were no other significant improvements in NIBUT and Schirmer’s 1. Rexon-Eye also improved subjective DED scores in 41.7% of eyes without any adverse effects. There were no significant differences in the other DED measures between both groups. There were also no significant differences seen in the tear cytokine profile between both groups 2 months after treatment.

We found that there was significant improvement in the inferior cornea staining in the eyes treated with Rexon-Eye, compared to placebo. In our sub-analysis of corneal fluorescein staining outcomes in the full power treatment group, we observed that the participants with more severe baseline staining (CCLRU total score of >9.8) had a significantly greater reduction of corneal staining than the participants starting with less severe staining. This novel observation could suggest that the amount of benefit in improvement of corneal staining derived from this treatment may be correlated to the severity of initial corneal staining. Overall, it was encouraging to see that patients were more severe DED benefited more with Rexon-Eye compared to those with better cornea staining, suggesting that epithelial disease may be improved with such treatment. More future work would still be needed to confirm such a finding. However, did not find significant differences in the other objective DED parameters of NIBUT, Schirmer’s 1 and corneal fluorescein staining between the treatment and control groups (all with moderate to severe DED).

Our results that Rexon-Eye could improve subjective DED measures are supported by earlier studies. Trivli et al. (13) and Pedrotti (5) both showed in non-randomized interventional cohort studies that DED patients experienced a subjective benefit in the Ocular Surface Disease Index (OSDI) score post-Rexon, where Ferrari et al. (14) reported that in patients affected by Meibomian gland dysfunction, Rexon-Eye improved total OSDI scores by 37%.

Although these studies did not use a parallel control group, other different forms of transcutaneous electrical stimulation (TES) therapy did show similar positive results. For instance, Cai et al. reported in a randomized controlled trial of 104 eyes in 52 DED patients that those treated with both TES therapy via the Huatuo SDZ-II electrical stimulator and sodium hyaluronate eyedrops had significantly better OSDI scores compared with patients treated with sodium hyaluronate eyedrops alone (22). Han et al. demonstrated in a prospective randomized clinical trial of 24 patients for prevention of dry eye after PRK, that patients who had TES via a goggle-like device called Nu Eyne 01 (Nu Eyne Co., Ltd., Seoul, Republic of Korea) had significantly lower eye pain intensity compared to control group by 65% at the first week post-PRK due to an improvement in DED-related corneal nerve damage, although OSDI and SPEED II questionnaire scores were similar between the 2 groups at 3 months post-surgery (23). Sivanesan et al. found in a small pilot study that the use of TES in the stimulation of the trigeminal nerve achieved a short-term reduction in DED-related chronic ocular pain by 57% and photophobia by 28% (24). Friedman et al. revealed an improvement in OSDI scores in a non-randomized clinical trial, using neurostimulation of the nasal sensory nerves via the nasolacrimal pathway, however, there were concerns of hygiene and patient discomfort (25).

In our study, we found that both full treatment and control arms had improvement in SPEED scores. Unlike other studies, all participants were continued on the pre-existing treatment including immunosuppressive eyedrops, so it would be harder to demonstrate improvement of SPEED and objective clinical parameters. A total of 62% of eyes in the control group also had improvement in SPEED scores too; we are uncertain if the improvement in the control group is related to better medication compliance and outcomes, since clinical trial patients are monitored more closely than routine clinic patients. Nonetheless, our results lend support to the potential adjunctive use of the Rexon- Eye in treating and improving subjective DED in our moderate to severe Asian DED patients in the armamentarium of treatment for dry eyes, especially those with recalcitrant symptoms.

In DED, the degree of ocular surface inflammation is an important quantifiable parameter for diagnosis and monitoring of the disease before and after treatments. Tear collection followed by cytokine determination if a less invasive measure of documenting inflammation compared to other methods such as impression cytology or tissue biopsy (26–28). Earlier studies have shown that alterations of the tear cytokine profiles occur in DED patient, with elevated proinflammatory cytokine levels such as IL-1, IL-6, IL-8, and TNF-α shown to be strongly correlated with dry eye markers (29), leading to ocular surface damage and goblet cell reduction (30). Topical anti-inflammatory treatments such as ciclosporin (31) or methylprednisolone (32) are effective in reducing the levels of inflammatory cytokine levels in tears. In our study, we found that 11 different tear cytokine levels did not differ significantly pre- and post-Rexon-Eye treatment. To the best of our knowledge, this study is the first to analyze tear cytokine changes with Rexon-Eye treatment. However, we postulate that the possible reason for our observation of the tear film cytokine levels could be attributed to the time-frame that the subjects’ tear secretion was measured at; which was at 2 months after cessation of Rexon-Eye therapy. We are unsure if the effect of Rexon-Eye on the tear film and ocular surface inflammation may be more transient than expected and not last beyond 2 months. Trivli et al. reported earlier that Rexon-Eye treatment was able to show a 75% reduction in tear MMP-9-positive patients compared to pre-treatment levels (13).

The exact mechanisms underlying the benefits of Rexon-Eye on DED remain unclear. Previous studies have hypothesized that in transcutaneous application of electrical stimulation, electric fields can hasten cell migration during the wound healing process by improving cell directionality (33). Different groups of cells, including neural crest cells (34, 35), fibroblasts (33), neurons (36, 37), and CECs (38–40), respond to electric fields by directed migration or directed growth *in vitro*. This has been shown to increase the rate of cornea healing in cornea abrasions in a vivo model of corneal wound healing in rabbits (10). It is also hypothesized that QMR can stimulate the lacrimal system, reactivate the lacrimal and meibomian gland tissue and benefit the ocular annexes (5).

Our study has several limitations. First, our study has a relatively small sample size and did not have true observer masking because some participants that underwent QMR with the full treatment power may experience a heating sensation. We did not categorize the different subtypes of DED in our subjects, although Trivli et al. suggested that Rexon-Eye could improve both inflammation and meibomian gland quality (13). We also did not have any immediate or intermediate visits 1 month or longer-term after the treatment, and did not measure tear film lipid layer or osmolarity at our timepoints. Furthermore, single timepoints differences across the various DED parameters between the 2 groups are difficult to account for, and could be due to individual differences within each group at baseline.

Our study’s strengths include the use of detailed subjective and objective parameters, including the SPEED and QUEST questionnaires, the first use of the multiplex cytokine assay to evaluate the effects of Rexon-Eye on DED. A total of 83% of subjects found the Rexon-Eye treatment pleasant, compared to 50% in the control treatment group, and 41.2% felt that the Rexon-Eye treatment was more effective than other DED treatments and had improved DED symptoms compared to before treatment. Future studies with larger sample sizes and longer follow-up times would be useful in confirming our findings. Because of the apparent greater efficacy of corneal epithelial healing in cases with greater staining, it is also worth evaluating the use of Rexon-Eye in persistent corneal epithelial defects.

In conclusion, in Asian recalcitrant dry eyes treated with Rexon-Eye, inferior cornea staining showed significant improvement compared to placebo, and eyes with greater cornea staining at baseline achieved a greater improvement in staining. There were no other significant improvements in NIBUT and Schirmer’s 1. Rexon-Eye also improved subjective DED scores in 41.7% of eyes without any adverse effects. Other objective DED markers were not significantly altered after therapy. The tear cytokine levels were not significantly different between both treatment and control groups 2 months after the use of Rexon-Eye. Rexon-Eye as an adjunctive therapy in Asian recalcitrant moderate to severe DED is promising especially for a specific subgroup of more advanced DED subjects, but the characterization of this subgroup needs further evaluation. In future, we could focus on patients with severe and poorly responsive DED and evaluate changes in DED parameters earlier on at 1-month from the treatment.

## Data availability statement

The raw data supporting the conclusions of this article will be made available by the authors, without undue reservation.

## Author contributions

LT, VF, and Y-CL have made a substantial contribution to the concept or design of the article, and the acquisition, analysis, or interpretation of data for the article. All authors drafted the article or revised it critically for important intellectual content.

## References

[1] Craig J Nichols K Akpek E Caffery B Dua H Joo C Definition and classification report. *Ocul Surf.* (2017) 15:276–83. 10.1016/j.jtos.2017.05.008 28736335

[2] Agarwal P Craig J Rupenthal I. Formulation considerations for the management of dry eye disease. *Pharmaceutics.* (2021) 13:207. 10.3390/pharmaceutics13020207 33546193 PMC7913303

[3] O’Neil E Henderson M Massaro-Giordano M Bunya V. Advances in dry eye disease treatment. *Curr Opin Ophthalmol.* (2019) 30:166. 10.1097/ICU.0000000000000569 30883442 PMC6986373

[4] Shen Lee B Toyos M Karpecki P Schiffbauer J Sheppard J. Selective pharmacologic therapies for dry eye disease treatment: efficacy, tolerability, and safety data review from preclinical studies and pivotal trials. *Ophthalmol Ther.* (2022) 11:1333–69. 10.1007/s40123-022-00516-9 35608780 PMC9253213

[5] Pedrotti E Bosello F Fasolo A Frigo A Marchesoni I Ruggeri A Transcutaneous periorbital electrical stimulation in the treatment of dry eye. *Br J Ophthalmol.* (2017) 101:814–9. 10.1136/bjophthalmol-2016-308678 27660329

[6] Maschio M Canato M Pigozzo F. Biophysical effects of high frequency electrical field (4 MHz) on muscle fibers in culture. *Basic Appl Myol.* (2009) 19:49–56.

[7] Song B Gu Y Pu J Reid B Zhao Z Zhao M. Application of direct current electric fields to cells and tissues in vitro and modulation of wound electric field in vivo. *Nat Protoc.* (2007) 2:1479–89. 10.1038/nprot.2007.205 17545984

[8] Haastert-Talini K Grothe C. Electrical stimulation for promoting peripheral nerve regeneration. *Int Rev Neurobiol.* (2013) 109:111–24. 10.1016/B978-0-12-420045-6.00005-5 24093609

[9] Song B Zhao M Forrester J McCaig C. Nerve regeneration and wound healing are stimulated and directed by an endogenous electrical field in vivo. *J Cell Sci.* (2004) 117:4681–90. 10.1242/jcs.01341 15371524

[10] Ghaffarieh A Ghaffarpasand F Dehghankhalili M Honarpisheh N Nirumandi S Tanideh N. Effect of transcutaneous electrical stimulation on rabbit corneal epithelial cell migration. *Cornea.* (2012) 31:559–63. 10.1097/ICO.0b013e31823f8b2a 22333665

[11] Wenjin W Wenchao L Hao Z Feng L Yan W Wodong S Electrical stimulation promotes BDNF expression in spinal cord neurons through Ca(2+)- and Erk-dependent signaling pathways. *Cell Mol Neurobiol.* (2011) 31:459–67. 10.1007/s10571-010-9639-0 21259048 PMC11498367

[12] Sella S Adami V Amati E Bernardi M Chieregato K Gatto P In-vitro analysis of quantum molecular resonance effects on human mesenchymal stromal cells. *PLoS One.* (2018) 13:e0190082. 10.1371/journal.pone.0190082 29293552 PMC5749755

[13] Trivli A Karmiris E Dalianis G Ruggeri A Terzidou C. Evaluating the efficacy of quantum molecular resonance (QMR) electrotherapy in mixed-type dry eye patients. *J Opt.* (2022) 16:128–34. 10.1016/j.optom.2022.06.003 35851496 PMC10104789

[14] Ferrari G Colucci A Barbariga M Ruggeri A Rama P. High frequency electrotherapy for the treatment of meibomian gland dysfunction. *Cornea.* (2019) 38:1424–9. 10.1097/ICO.0000000000002063 31356415

[15] Fraccalvieri M Salomone M Di Santo C Ruka E Morozzo U Bruschi S. Quantum molecular resonance technology in hard-to-heal extremity wounds: histological and clinical results. *Int Wound J.* (2017) 14:1313–22. 10.1111/iwj.12805 28857452 PMC7950067

[16] Trobinger V. *Esperienza clinica nell’impiego del dispositivo Rexon-Age.* (2009). Available online at: http://teleamedical.com/telea2013/wpcontent/uploads/fisioterapia-04.pdf (accessed March 13, 2023).

[17] Lopresti M Tomba A Caserta A Di Domenica F. Studio clinico sull’efficacia della risonanza quantica molecolare nel trattamento dell’edema post-chirurgico in pazienti sottoposti a intervento di artroprotesi di ginocchio. *Arch Ortope Reumatol.* (2011) 122:34–5. 10.1007/s10261-011-0013-7

[18] Terry R Schnider C Holden B Cornish R Grant T Sweeney D CCLRU standards for success of daily and extended wear contact lenses. *Opt Vis Sci.* (1993) 70:234–43. 10.1097/00006324-199303000-00011 8483586

[19] Ngo W Situ P Keir N Korb D Blackie C Simpson T. Psychometric properties and validation of the standard patient evaluation of eye dryness questionnaire. *Cornea.* (2013) 32:1204–10. 10.1097/ICO.0b013e318294b0c0 23846405

[20] Lee R Yeo S Aung H Tong L. Agreement of noninvasive tear break-up time measurement between Tomey RT-7000 auto refractor-Keratometer and oculus Keratograph 5M. *Clin Ophthalmol.* (2016) 10:1785–90. 10.2147/OPTH.S110180 27695283 PMC5033501

[21] Willems B Tong L Minh T Pham N Nguyen X Zumbansen M. Novel cytokine multiplex assay for tear fluid analysis in Sjogren’s syndrome. *Ocul Immunol Inflamm.* (2021) 29:1639–44. 10.1080/09273948.2020.1767792 32657632

[22] Cai M Zhang J. Effectiveness of transcutaneous electrical stimulation combined with artificial tears for the treatment of dry eye: a randomized controlled trial. *Exp Ther Med.* (2020) 20:175. 10.3892/etm.2020.9305 33093910 PMC7571363

[23] Han G Yoo Y Shin E Park J Park Y Kim P Transcutaneous electrical stimulation for the prevention of dry eye disease after photorefractive keratectomy: randomized controlled trial. *Ophthalmol Sci.* (2022) 3:100242. 10.1016/j.xops.2022.100242 36685712 PMC9853365

[24] Sivanesan E Levitt R Sarantopoulos C Patin D Galor A. Noninvasive electrical stimulation for the treatment of chronic ocular pain and photophobia. *Neuromodulation.* (2018) 21:727–34. 10.1111/ner.12742 29283468 PMC6023783

[25] Friedman N Butron K Robledo N Loudin J Baba S Chayet A. A nonrandomized, open-label study to evaluate the effect of nasal stimulation on tear production in subjects with dry eye disease. *Clin Ophthalmol.* (2016) 10:795–804. 10.2147/OPTH.S101716 27217719 PMC4862385

[26] Tong L Beuerman R Simonyi S Hollander D Stern M. Effects of punctal occlusion on clinical signs and symptoms and on tear cytokine levels in patients with dry eye. *Ocul Surf.* (2016) 14:233–41. 10.1016/j.jtos.2015.12.004 26774908

[27] Tong L Wong T Cheng C. Level of tear cytokines in population-level participants and correlation with clinical features. *Cytokine.* (2018) 110:452–8. 10.1016/j.cyto.2018.05.013 29803660

[28] Le Guezennec X Quah J Tong L Kim N. Human tear analysis with miniaturized multiplex cytokine assay on “wall-less” 96-well plate. *Mol Vis.* (2015) 21:1151–61. 26539027 PMC4605753

[29] Roda M Corazza I Bacchi Reggiani M Pellegrini M Taroni L Giannaccare G Dry eye disease and tear cytokine levels-a meta-analysis. *Int J Mol Sci.* (2020) 21:3111. 10.3390/ijms21093111 32354090 PMC7246678

[30] Yamaguchi T. Inflammatory response in dry eye. *Invest Ophthalmol Vis Sci.* (2018) 59:DES192–9. 10.1167/iovs.17-23651 30481826

[31] Periman L Mah F Karpecki PMA. Review of the mechanism of action of cyclosporine a: the role of cyclosporine a in dry eye disease and recent formulation developments. *Clin Ophthalmol.* (2020) 14:4187–200. 10.2147/OPTH.S279051 33299295 PMC7719434

[32] Lee J Min K Kim S Kim E Kim T. Inflammatory cytokine and osmolarity changes in the tears of dry eye patients treated with topical 1% methylprednisolone. *Yonsei Med J.* (2014) 55:203–8. 10.3349/ymj.2014.55.1.203 24339308 PMC3874929

[33] Stump R Robinson K. Xenopus neural crest cell migration in an applied electrical field. *J Cell Biol.* (1983) 97:1226–33. 10.1083/jcb.97.4.1226 6619192 PMC2112593

[34] Zhao M Penninger J Isseroff R. Electrical activation of wound-healing pathways. *Adv Skin Wound Care.* (2010) 1:567–73. 22025904 10.1089/9781934854013.567 PMC3198837

[35] Nuccitelli R Erickson C. Embryonic cell motility can be guided by physiological electric fields. *Exp Cell Res.* (1983) 147:195–210. 10.1016/0014-4827(83)90284-7 6617761

[36] Erickson C Nuccitelli R. Embryonic fibroblast motility and orientation can be influenced by physiological electric fields. *J Cell Biol.* (1984) 98:296–307. 10.1083/jcb.98.1.296 6707093 PMC2112998

[37] Hinkle L McCaig C Robinson K. The direction of growth of differentiating neurones and myoblasts from frog embryos in an applied electric field. *J Physiol.* (1981) 314:121–35. 10.1113/jphysiol.1981.sp013695 7310685 PMC1249421

[38] Jaffe L Poo M. Neurites grow faster towards the cathode than the anode in a steady field. *J Exp Zool.* (1979) 209:115–28. 10.1002/jez.1402090114 490126

[39] Soong H Parkinson W Bafna S Sulik G Huang S. Movements of cultured corneal epithelial cells and stromal fibroblasts in electric fields. *Invest Ophthalmol Vis Sci.* (1990) 31:2278–82. 2242993

[40] Zhao M Agius-Fernandez A Forrester J McCaig C. Orientation and directed migration of cultured corneal epithelial cells in small electric fields areserum dependent. *J Cell Sci.* (1996) 109:1405–14. 10.1242/jcs.109.6.1405 8799828

