# Supplementary material for: Quantum molecular resonance electrotherapy (Rexon-Eye) for recalcitrant dry eye in an Asian population
Source: Front Med (Lausanne). 2023 Sep 12;10:1209886. doi: 10.3389/fmed.2023.1209886 (PMC10523309; doi:10.3389/fmed.2023.1209886)
Supplement: Supplementary file 1 [file Presentation_1.pdf]

Supplementary Appendix 1

QUEST and Acceptability and Satisfaction Questionnaire

		Notes
The situation compared to before treatment is now:	☐ better<br>☐ same<br>☐ worse	
The situation compared to end of treatment is now:	☐ better<br>☐ same<br>☐ worse	Indicate what treatment was used after end of treatment
The situation compared to 1 month after end of treatment is now:	☐ better<br>☐ same<br>☐ worse	
During last week I experienced these eye symptoms:	☐ none<br>☐ pain<br>☐ burning<br>☐ sand<br>☐ dryness<br>☐ photophobia<br>☐ other (specify)	
During last week I used tear substitutes during the day:	☐ yes<br>☐ no	Indicate no. of times eyedrops used per day
During last week I used tear substitutes during the evening / night:	☐ yes<br>☐ no	
In the last 2 months I had eye problems other than dry eye:	☐ yes<br>☐ no	
In the last 2 months I used tear substitutes:	☐ yes<br>☐ no	
In the last 2 months I tried to suspend using tear substitutes	☐ yes, and it worked<br>☐ yes, but it did not work and I resumed using tear substitutes<br>☐ no	

Compared to before treatment my eyes now produce more tears	☐ yes<br>☐ no<br>☐ don’t know	
Compared to before treatment my eyes now are more wet (tears are more effective):	☐ yes<br>☐ no<br>☐ don’t know	
Compared to before treatment my eyes now feel better:	☐ yes<br>☐ no<br>☐ don’t’ know	
Compared to before treatment I have reduced the use of tear substitutes:	☐ yes<br>☐ no<br>☐ don’t know	
Overall, I judge the treatment as pleasant:	☐ yes<br>☐ no<br>☐ neutral	
Overall, I judge the treatment as effective:	☐ yes<br>☐ no<br>☐ neutral	
Overall, I judge the treatment as durable:	☐ yes<br>☐ no<br>☐ neutral	
Overall, I judge the treatment, with respect to other treatments I tried:	☐ better<br>☐ same<br>☐ worse	
