# Supplementary material for: Quantum molecular resonance electrotherapy (Rexon-Eye) for recalcitrant dry eye in an Asian population
Source: Front Med (Lausanne). 2023 Sep 12;10:1209886. doi: 10.3389/fmed.2023.1209886 (PMC10523309; doi:10.3389/fmed.2023.1209886)
Supplement: Supplementary file 2 [file Data_Sheet_1.docx]

**Supplementary 1 – *Tear Cytokine Elution and Analysis***

Without prior anesthesia, Schirmer strips were folded between the notch and the top of the strip and hooked over the lower eyelid of patients without contact with the cornea for 5 minutes. After removal, the wetting length was recorded and the strip was placed into a 1.5ml reaction tube and stored at -80 °C. After thawing the strips were cut at the wetting front and the non-wet portion was discarded. The wet part was placed in a 1.5ml reaction tube with the addition of ten times excess of Assay Diluent 2 (AD2, AYOXXA, Germany). The tear fluid volume was calculated by multiplying the wetting length in mm by 2/3. To elute the tear fluid the tube was placed in a thermomixer for 30 minutes at 20 °C at 1000rpm. Subsequently the strip was clamped into the lid of the tube and spin-dried in a table-top tube centrifuge at 6000 rpm for 1 minute. The dried paper was discarded and the collected liquid kept on ice for analysis with the AYOXXA LUNARIS system. 1 μl of the fluid was used for the assessment of total protein content with a Nanodrop photospectrometer. 5 μl of the elute were pipetted into the wells of the AYOXXA Biochip. Duplicate measurement was done for all samples. Antibody-coated beads are pre-deposited in spatially separated micro-cavities on the planar LUNARIS BioChip. Briefly, after 3 hours of incubation at room-temperature, the wells were incubated for 1 hour with capture antibody solution and subsequently 30 minutes with streptavidin bound phycoerythrine solution. Thorough washing steps were performed in between incubation periods. After drying the fluorescent intensity of all microbeads of all microbeads was automatically detected microscopically with the AYOXXA Reader. Median fluorescent intensities (MFIs) were calculated from all beads per analyte per well. Using the standard curve calculated with a four parameter logistic curve fit from the mean MFI values of triplicates of the standard dilution series, the sample MFI are translated into analyte concentrations. All sample concentrations below the limit of detection (LoD) were given a proximity value of LoD/2. All sample concentrations above the upper limit of quantification (UloQ) were given a proximity value of equal to the UloQ. To account for the variation of physiological tear fluid dilution, all sample concentrations were normalised for their total protein concentration and given as pigo-gram per milligram of total protein (pg/ml).
